# Tuftsin Promotes an Anti-Inflammatory Switch and Attenuates Symptoms in Experimental Autoimmune Encephalomyelitis

**DOI:** 10.1371/journal.pone.0034933

**Published:** 2012-04-17

**Authors:** Muzhou Wu, Jillian C. Nissen, Emily I. Chen, Stella E. Tsirka

**Affiliations:** 1 Department of Pharmacological Sciences, School of Medicine, Stony Brook University, Stony Brook, New York, United States of America; 2 Program in Neuroscience, School of Medicine, Stony Brook University, Stony Brook, New York, United States of America; 3 Program in Molecular and Cellular Pharmacology, School of Medicine, Stony Brook University, Stony Brook, New York, United States of America; 4 Stony Brook University Proteomics Center, School of Medicine, Stony Brook University, Stony Brook, New York, United States of America; Institute Biomedical Research August Pi Sunyer (IDIBAPS) - Hospital Clinic of Barcelona, Spain

## Abstract

Multiple sclerosis (MS) is a demyelinating autoimmune disease mediated by infiltration of T cells into the central nervous system after compromise of the blood-brain barrier. We have previously shown that administration of tuftsin, a macrophage/microglial activator, dramatically improves the clinical course of experimental autoimmune encephalomyelitis (EAE), a well-established animal model for MS. Tuftsin administration correlates with upregulation of the immunosuppressive Helper-2 Tcell (Th2) cytokine transcription factor GATA-3. We now show that tuftsin-mediated microglial activation results in shifting microglia to an anti-inflammatory phenotype. Moreover, the T cell phenotype is shifted towards immunoprotection after exposure to tuftsin-treated activated microglia; specifically, downregulation of pro-inflammatory Th1 responses is triggered in conjunction with upregulation of Th2-specific responses and expansion of immunosuppressive regulatory T cells (Tregs). Finally, tuftsin-shifted T cells, delivered into animals via adoptive transfer, reverse the pathology observed in mice with established EAE. Taken together, our findings demonstrate that tuftsin decreases the proinflammatory environment of EAE and may represent a therapeutic opportunity for treatment of MS.

## Introduction

Multiple sclerosis (MS) is a chronic debilitating autoimmune disease in which T cells, microglia and macrophages aberrantly attack the myelin sheath that protects nerve fibers in the brain and spinal cord. The symptoms of MS vary from sensory defects, such as blurry vision, to loss of balance, muscle weakness, and paralysis [1]. Experimental autoimmune encephalomyelitis (EAE) is an induced autoimmune disease used as one of the animal models to study MS. During EAE, activated T cells, which are normally absent from the central nervous system (CNS), infiltrate the CNS through the compromised blood-brain barrier (BBB). The activated T cells initiate an inflammatory cascade that generates cytokines and chemokines, and attack the myelinated neurons causing demyelination in the CNS. Resident microglia also undergo activation and trigger the recruitment of peripheral macrophages, which release cytokines and chemokines that propagate disease progression [2]–[5] and subsequent recovery.

Microglia are the immune-competent cells that reside in the CNS. During MS and EAE, they become activated and contribute to the inflammatory process through several mechanisms, including phagocytosis and production of various factors such as cytokines, free radicals, and metalloproteinases [6]. However, the effects of microglia are not solely deleterious; microglia also produce anti-inflammatory cytokines, such as TGFβ and IL10 [7], which are associated with inhibition or prevention of EAE. The timing and strength of these protective and neurotoxic outputs determine which overall effect predominates.

T cells undertake the primary role in modulating the outcome of MS/EAE. Naïve T cells can differentiate into helper (Th) and regulatory cells (Tregs). There are three subsets of T helper cells: Th1, Th2 [8] and the more recently described Th17 cells [9]. Th1 cells produce proinflammatory cytokines (e.g. TNFalpha) and mediate proinflammatory responses during MS/EAE, whereas Th2 cells secrete anti-inflammatory cytokines [e.g. interleukin-4 (IL4), -10 (IL10), and -13 (IL13)] and participate in prevention or remission of MS/EAE. Th17 cells produce interleukin-17 (IL17) and play a pathogenic role in inducing autoimmune tissue inflammation [10]–[12]. In the presence of TGFβ, naive T cells become Tregs, which express FOXP3 and suppress immune system activation [11], [13]–[16].

Tuftsin is a naturally occurring tetrapeptide (threonine-lysine-proline-arginine) that was described originally as a phagocytosis-stimulating factor derived from the proteolytic processing of IgG [17]. Tuftsin promotes phagocytic activity for cells of monocytic origin, such as neutrophils, macrophages and microglia, all of which are thought to express tuftsin receptors. Tuftsin or tuftsin-like peptides also exert other stimulatory effects, including enhanced migration/chemotaxis and antigen presentation, and can affect T-cell function as well [18]. Moreover, tuftsin may have direct effects on the nervous system, including induction of analgesia [19] and inhibition of axonal regeneration [20].

Previous work in our laboratory revealed that modification of the status of microglia affected the timing and symptoms of EAE [21]. In particular, the microglial activator tuftsin, which readily crosses into the CNS [22], decreased the severity of EAE symptoms and drastically improved recovery in wild-type mice. Real-time PCR data revealed that wild-type EAE mice exhibited prevalent T-bet expression, which is a transcription factor that promotes Th1 lineage development and cytokine production. Conversely, tuftsin infusion into wild-type mice subjected to EAE resulted in increased GATA3 expression, which is a transcription factor that drives Th2 lineage development and release of anti-inflammatory cytokines. In this study, we used both *in culture* and *in vivo* methods to investigate the mechanism through which tuftsin modulates the immune response in EAE. Our results show that *in culture*, modulating microglial activity with tuftsin affects T cell fate by downregulating the Th1-proinflammatory responses and upregulating anti-inflammatory cytokines. *In vivo*, tuftsin infusion promotes dominance of a Th2 phenotype with increases in anti-inflammatory cytokines and the emergence of markers characteristic of immunosuppressive Tregs. Finally, adoptive transfer of tuftsin-modulated T cells reverses clinical signs of disease in mice with established EAE, which suggests promising therapeutic opportunities.

## Materials and Methods

### Animals

C57BL/6 mice were bred in-house under specific pathogen-free conditions [Division of Laboratory Animal Resources at the State University of New York (SUNY) Stony Brook], controlled for temperature (21°C), and maintained under a 12-hour light/dark cycle. Access to food and water was ad libitum. Adult (6- to 8-week-old) female mice were used in all experiments.

### MOG peptide

MOG35-55 peptide (MEVGWYRSPFSRVVHLYRNGK) was synthesized by Quality Controlled Biochemicals and purified using reverse-phase (C18) HPLC.

### Induction of EAE with MOG_35–55_ peptide

EAE was induced as described previously [21], [23], [24] by subcutaneous injection into the mouse flank on day 0 with 300 µg of MOG_35–55_ peptide thoroughly emulsified in complete Freund's adjuvant (CFA) containing 500 µg of heat-inactivated Mycobacterium tuberculosis (Difco, Detroit, MI). One week later (day 7), the mice were boosted with 300 µg of MOG_35–55_ peptide subcutaneously in the other flank. 500 ng Pertussis toxin (List Biologicals, Campbell, CA) in 200 µl of PBS was injected intraperitoneally on days 0 and 2.

### Evaluation of EAE symptoms

After immunization with MOG, mice were observed and weighed daily blindly. Symptom severity was scored on a scale of 0 to 5 with graduations of 0.5 for intermediate symptoms. The score is defined as follows [25]: 0, no detectable symptoms; 1, loss of tail tone; 2, hindlimb weakness or abnormal gait; 3, complete paralysis of the hindlimbs; 4, complete hindlimb paralysis with forelimb weakness or paralysis; 5, moribund or death.

### Time-controlled Drug Delivery

Alzet mini-osmotic pumps (Durect, Cupertino, CA) were used to ensure time-controlled drug delivery. 14-day pumps (rate of infusion 0.25 µl/hr, 100 µl total volume) are filled with either PBS or 500 µM tuftsin (American Peptide Company, Sunnyvale, CA) and incubated at 37°C overnight before using. Adult female C57Bl6 mice (6–10 weeks old) were deeply anesthetized, and pumps were implanted subcutaneously in the back of the mice for on day 1 after MOG immunization. Pumps were replaced with fresh 14-day pumps on day 15 and maintained for the duration of the experiment.

### Eriochrome Cyanine stain and myelin quantification

Eriochrome cyanine (EC) staining was used to visualize myelin in the lumbar region of the spinal cord, as we have previously described [26]. In brief, spinal cord sections previously stored at −80°C were air-dried for 1 hour at room temperature and a second hour at 37°C in a dry incubator. After incubation with acetone for 5 minutes, the slides were air-dried for 30 minutes and then stained in EC solution (0.2% eriochrome cyanine RS (Sigma), 0.5% H_2_SO_4_ (Sigma), 10% iron alum (Sigma) in distilled water for 30 minutes, differentiated in 5% iron alum (Sigma) for 10 minutes, and placed in borax-ferricyanide solution (1% borax (Sigma), 1.25% potassium ferricyanide (Sigma), in distilled water) for 5 minutes. The slides were then dehydrated through graded ethanol solutions and coverslipped using Permount (Fisher Scientific, NJ, USA). These were then visualized on a Nikon Eclipse E600 microscope. Multiple images of several sections from the same animal of the ventral white matter taken at 40× magnification were quantified for their demyelinated (unstained) area using ImageJ software. When fluoromyelin was used the quantification derived from sections from the lumbar spinal cord using ImageJ. All white matter area was selected and average intensity (total intensity/area) was generated as (Ave I_whitematter_); then all grey matter area was selected (as background), the average intensity (total intensity/area) was generated as (Ave I_greymatter_). Then the final value was (Ave I_whitematter_)−(Ave I_greymatter_). The 3 different timepoints were chosen because they represent three different stages during disease development and immune cell response: early stage, peak and recovery.

### Immunofluorescence

Mice were transcardiacally perfused using 4% paraformaldehyde/PBS and the spinal cords removed and post-fixed in 4% paraformaldehyde/PBS for 1 hour at room temperature followed by 30% sucrose dehydration at 4°C overnight. The spinal cords were embedded in Tissue-Tek (Miles, Elkhart, IN) optimal cutting temperature compound, frozen on dry ice, and stored at 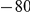
°C until use. Cross sections (20 µm) were cut on a cryostat (Leica, Nussloch, Germany) at 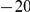
°C.

Spinal cord sections were blocked in serum of the host of the secondary antibody [5% serum in PBS-T (0.5% TritonX-100 in PBS)], and then incubated overnight at 4°C in rabbit anti-mouse Iba1 (Wako, Japan) at a 1∶500 dilution in PBS to detect both resting and activated microglia/macrophages. After washing in PBS, sections were incubated with fluorescence-conjugated (FITC or Texas Red) goat anti-rabbit secondary antibody for 1 hour at room temperature, washed 3 times 10 minutes each with PBS, and mounted using Fluoromount-G (Southern Biotech, USA).

### Primary T cells culture from mouse spleen

The T cell negative isolation kit (Invitrogen) was used to retrieve primary T cells from mouse spleens. Briefly, a mixture of monoclonal antibodies against unwanted cells (B cells, NK cells, monocytes, dendritic cells, CD8^+^ T cells, erythrocytes and granulocytes) was added to spleen cells. T cells were isolated by removing the antibody-labeled cells using mouse depletion Dynabeads and a Dynal MPC. The CD4^+^ T cells were stimulated with plate-bound anti-CD3 (10 µg/ml, BD Biosciences) antibody, serving as ‘artificial APCs’ [27]. Cells were washed and restimulated with anti-CD3 (5 µg/ml) before cytokine analysis, as in [28].

### Measurement of cytokine production

Purified T cells (2.5×10^5^ cells well) were stimulated with 0.1–100 µg/ml MOG_35–55_ and/or 0.1–1 µg/ml plate-bound anti-CD3/soluble anti-CD28 in complete DMEM medium at 37°C. Culture supernatants were harvested 48 h later and the levels of cytokines were determined via ELISA. To measure levels of TNFα/IL10 in the culture medium, the OptEIA Set Mouse TNF/IL10 kits from BD Biosciences were used according to manufacturer's instructions. Briefly, the wells of a Nunc MaxiSorp 96-well plate were coated overnight with the appropriate dilution of capturing antibody in coating buffer (0.2 M sodium phosphate buffer pH 6.5). The plate was washed three times with PBS-T (PBS, 0.05% Tween-20) and blocked with Assay Diluent for 1 hr at RT. After washing as before, 100 µl of sample or cytokines standard prepared in assay diluent were added followed by 2 h incubation at RT. After 5 washes with PBS-T, 100 µl of assay diluent containing biotin-conjugated detection antibody and Avidin-HRP reagent at the appropriate dilutions were added and incubated for 1 h at RT. Following 7 washes with PBS-T 100 µl of Substrate Solution was added to each well. After 30 min incubation in the dark, 50 µl of Stop Solution (2N H_2_SO_4_) were added and absorbance at 450 nm was read within 30 min on a SpectraMax microplate reader using the Softmax Pro software.

### Mixed cortical and primary microglia cultures

Tissue culture plates used for plating mixed cortical cultures were coated overnight at 4°C with 5 µg/ml poly-D-lysine (PDL, Sigma). Newborn (d0–d2) pups of wild-type mice were used to isolate cortical cells. The brains were removed, and cortices were freed from meninges, hippocampi and basal ganglia and kept in HBSS on ice. The cortical tissue was digested in 0.25% Trypsin/EDTA (Sigma) at 37°C for 20 min. The tissue was then triturated and filtered through a 40 µm cell strainer. The cell suspension was plated in the mixed cortical medium (DMEM, 10% FBS, 40 µg/ml Gentamycin).

The medium was changed 3 days after plating. Microglial cells were harvested 10 days after plating. Briefly, lidocaine was added directly to the culture medium at a final concentration of 1 mM and the culture left at room temperature for 15 min. The medium containing the floating microglia was collected and centrifuged at 500 g for 5 min, following which the cell pellet resuspended in microglia medium (DMEM, 1% FBS) and counted on a hemocytometer. The microglial cultures were >98% pure, as we have previously described [29]–[32].

### Primary Cortical Neuronal Cultures

Primary cortical neurons were cultured as previously described [33], [34]. Briefly, tissue culture plates were coated overnight at 4°C with 5 µg/ml poly-D-lysine (PDL, Sigma). Primary neuronal cultures were prepared from the cortices of embryonic day 14–15 pups. Briefly, mouse cortices were dissected and put in Hanks solution (HBSS), and gently trypsinized (0.25% trypsin in HBSS) at 37°C for 40 minutes and then triturated to form single cell suspensions. The cells were plated at a density of 100,000 cells/cm^2^ in Neurobasal medium with B27 supplements, 25 µM glutamate, 0.5 mM L-glutamine and 10 g/L gentamycin sulfate. AraC (10 µM) was added to the medium on day 2 of the culture to inhibit the growth of astrocytes. The neuronal cultures were >95% pure.

### PCR arrays

Mouse inflammatory cytokines and receptors arrays (SABioscience) were used on RNA samples isolated from T cells. Briefly, RNA was isolated from T cells after different treatments. cDNA was prepared from RNA samples using RT2 First Strand Kit (SABioscience). cDNA and RT-PCR master mix were added to 96-well PCR array plate. Thermal cycling was performed. Data was analyzed using PCR Array Data Analysis Web Portal provided by SABiosciences.

### RNA isolation and quantitative real-time PCR

RNA was isolated from cells using RNA-Bee (Tel-Test), by the manufacturer's protocol. To obtain cDNA, the High Capacity cDNA Reverse Transcription kit was used on a Veriti Thermocycler (Applied Biosystems). Amplification was performed on a StepOnePlus real-time PCR machine using a SYBR Green kit (Applied Biosystems). Primer sequences are as follows: GAPDH forward, 5′-GCACAGTCAAGGCCGAGAAT-3′; GAPDH reverse, 5′-GCCTTCTCCATGGTGGTGGA-3′; IL-10 forward, 5′-TGGCCACACTTGAGAGCTGC-3′; IL-10 reverse, 5′-TTCAGGGATGAAGCGGCTGG-3′; TNFα forward, 5′-ATGAGCACAGAAAGCATGATC-3′; TNFα reverse, 5′-TACAGGCTTGTCACTCGAATT-3′.

### Proteomic analysis of conditioned media

#### Sample Preparation

Samples for proteomic analysis were derived from serum- and supplement-free conditioned media, and were concentrated in Amicon Ultra-15, UltraCel 3 k columns (ThermoFisher) to acquire appropriate concentrations for digestion. Protein concentration was determined by the EZQ protein quantification assay (Invitrogen, CA) according to the manufacture suggested protocol.

#### Trypsin Digestion

50 µg of proteins from the conditioned media were diluted in 50 mM NH_4_HCO_3_ solution for trypsin digestion. Trypsin was added to each sample at a ratio of 1∶30 enzyme/protein along with 2 mM CaCl_2_ and incubated for 16 hours at 37°C. Following digestion, all reactions were acidified with 90% formic acid (2% final) to stop proteolysis. Then, samples were centrifuged for 30 minutes at 14,000 rpm to remove insoluble material. The soluble peptide mixtures were collected for LC-MS/MS analysis.

#### Multidimensional chromatography and tandem mass spectrometry

Peptide mixtures were pressure-loaded onto a 250 µm inner diameter (i.d.) fused-silica capillary packed first with 3 cm of 5 µm strong cation exchange material (Partisphere SCX, Whatman), followed by 3 cm of 10 µm C18 reverse phase (RP) particles (Aqua, Phenomenex, CA). Loaded and washed microcapillaries were connected *via* a 2 µm filtered union (UpChurch Scientific) to a 100 µm i.d. column, which had been pulled to a 5 µm i.d. tip using a P-2000 CO_2_ laser puller (Sutter Instruments), then packed with 13 cm of 3 µm C18 reverse phase (RP) particles (Aqua, Phenomenex, CA) and equilibrated in 5% acetonitrile, 0.1% formic acid (Buffer A). This split-column was then installed in-line with a NanoLC Eskigent HPLC pump. The flow rate of channel 2 was set at 300 nl/min for the organic gradient. The flow rate of channel 1 was set to 0.5 µl/min for the salt pulse. Fully automated 11-step chromatography runs were carried out. Three different elution buffers were used: 5% acetonitrile, 0.1% formic acid (Buffer A); 98% acetonitrile, 0.1% formic acid (Buffer B); and 0.5 M ammonium acetate, 5% acetonitrile, 0.1% formic acid (Buffer C). In such sequences of chromatographic events, peptides are sequentially eluted from the SCX resin to the RP resin by increasing salt steps (increase in Buffer C concentration), followed by organic gradients (increase in Buffer B concentration). The last chromatography step consists in a high salt wash with 100% Buffer C followed by acetonitrile gradient. The application of a 2.5 kV distal voltage electrosprayed the eluting peptides directly into a LTQ-Orbitrap XL mass spectrometer equipped with a nano-LC electrospray ionization source (ThermoFinnigan). Full MS spectra were recorded on the peptides over a 400 to 2000 m/z range by the Orbitrap, followed by five tandem mass (MS/MS) events sequentially generated by LTQ in a data-dependent manner on the first, second, third, and fourth most intense ions selected from the full MS spectrum (at 35% collision energy). Mass spectrometer scan functions and HPLC solvent gradients were controlled by the Xcalibur data system (ThermoFinnigan, San Jose, CA).

#### Database search and interpretation of MS/MS datasets

Tandem mass spectra were extracted from raw files, and a binary classifier - previously trained on a manually validated data set - was used to remove the low quality MS/MS spectra. The remaining spectra were searched against a UniProt mouse protein database released on May, 3^rd^ 2011 [35] and 124 common contaminant proteins. To calculate confidence levels and false positive rates, we used a decoy database containing the reverse sequences of the UniProt protein database appended to the target database [36], and the SEQUEST algorithm to find the best matching sequences from the combined database. SEQUEST searches were done through the Integrated Proteomics Pipeline (IP2, Integrated Proteomics Inc., CA) on Intel Xeon X5450 X/3.0 PROC processor clusters running under the Linux operating system. The peptide mass search tolerance was set to 50 ppm. No differential modifications were considered. A fully tryptic status was imposed on the database search.

The validity of peptide/spectrum matches was assessed in DTASelect2 [37] using SEQUEST-defined parameters, the cross-correlation score (XCorr) and normalized difference in cross-correlation scores (DeltaCN). The search results were grouped by charge state (+1, +2, and +3) and tryptic status (fully tryptic, half-tryptic, and non-tryptic), resulting in 9 distinct sub-groups. In each one of the sub-groups, the distribution of XCorr and DeltaCN values for (a) direct and (b) decoy database hits was obtained, and the two subsets were separated by quadratic discriminant analysis. Outlier points in the two distributions (for example, matches with very low Xcorr but very high DeltaCN were discarded. Full separation of the direct and decoy subsets is not generally possible; therefore, the discriminant score was set such that a false discovery rate of 1% was determined based on the number of accepted decoy database peptides. This procedure was independently performed on each data subset, resulting in a false positive rate independent of tryptic status or charge state. In addition, a minimum sequence length of 7 amino acid residues was required, and each protein on the final list was supported by at least two independent peptide identifications unless specified. These additional requirements – especially the latter - resulted in the elimination of most decoy database and false positive hits, as these tended to be overwhelmingly present as proteins identified by single peptide matches. After this last filtering step, the false discovery rate was reduced to below 1%. Relative fold difference between samples was derived using the spectral counting method [38], [39].

### Statistics

Statistical analysis was performed using one-way ANOVA followed by a Bonferroni-Dunn test for multiple comparisons within a group, or a two-tailed t-test for comparisons between groups as indicated in the figure legends; p<0.05 was considered significant and is marked by an (*); p<0.01 and p<0.001 were considered very significant and are marked by two (**); and three (***) respectively. All results are represented as average with error bars indicating the standard error of the mean. In all experiments, n refers to the number of animals used for each genotype or condition.

## Results

### Excitotoxic- and tuftsin-activated microglia shift the T cell phenotype towards immunosuppression *in culture*

We previously reported that tuftsin-infused mice exhibit an immune response that is shifted towards an anti-inflammatory phenotype [21]. We proposed that the immediate effect of tuftsin acceleration of microglial activation was to alter the relative abundance of T cell subsets (the T cell “phenotype”). However, it was also possible that tuftsin might exert a direct effect on T cells in this setting. We investigated these hypotheses using primary cell cultures.

**Figure 1:**
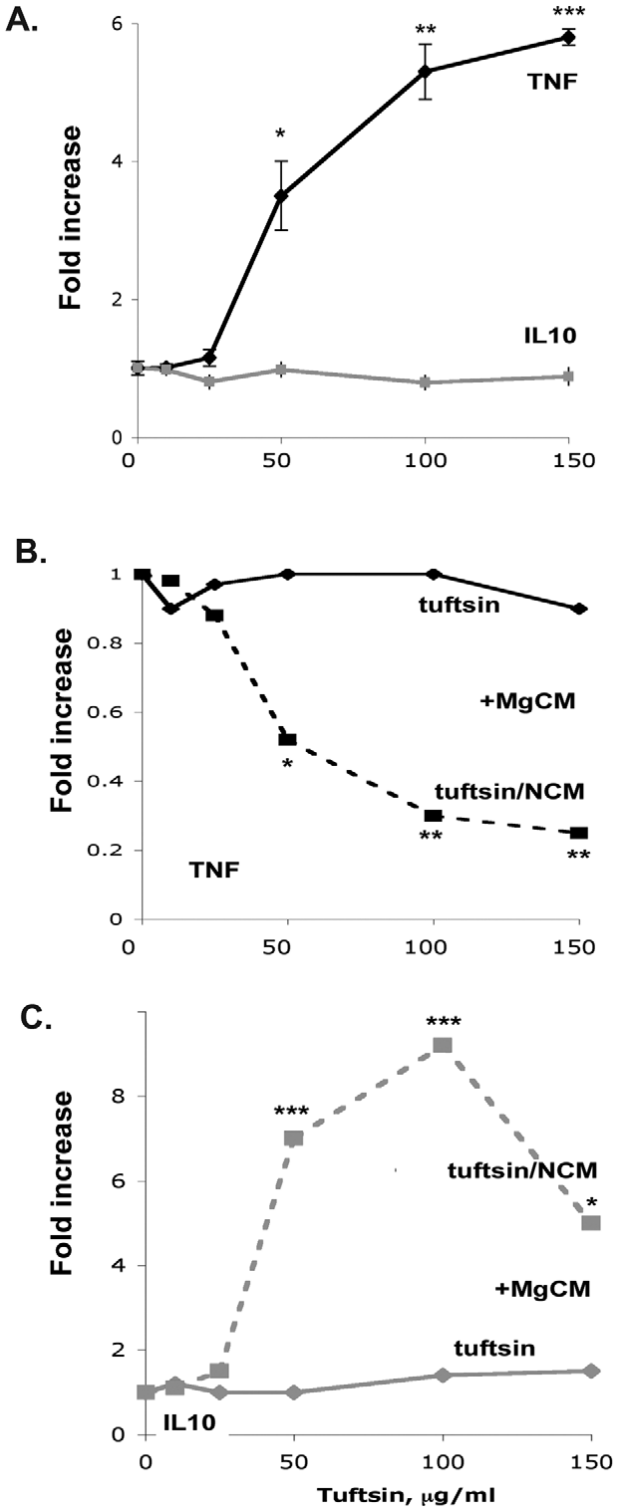
Cytokine production from direct or indirect exposure of T cells to tuftsin. (**A**) ELISA assays were performed to quantitate the proinflammatory cytokine TNFα and the anti-inflammatory cytokine IL10 as produced by splenic T cells exposed directly to varied concentrations of tuftsin, as described in Methods. n = 4, *, p<0.5; **, p<0.01; ***, p<0.001. When ELISA assays were performed to quantitate pro-inflammatory cytokine TNFα (**B**) and anti-inflammatory cytokine IL10 (**C**) production by T cells after treatment with microglial-conditioned medium (MgCM). The microglia were challenged either with tuftsin or with tuftsin and conditioned medium from neurons (NCM) that had been exposed to glutamate. n = 4, *, p<0.5; **, p<0.01; ***, p<0.001.

To test whether tuftsin has a direct effect on T cells, we isolated primary splenic T cells as described in Methods. A range of concentrations of tuftsin was added to individual cultures. 3 days later, supernatants from the T cell cultures were collected, and ELISA assays performed to quantitate the production of the Th1 cytokine tumor necrosis factor (TNFα) and the Th2 cytokine interleukin 10 (IL10). TNFα levels increased up to 6-fold (Fig. 1A), consistent with prior reports of T cells having a tuftsin receptor and producing TNFalpha in response to tuftsin stimulation [18], but there was no effect on production of IL10. Given that TNFα is a proinflammatory cytokine, these findings would not explain our prior results that tuftsin treatment *in vivo* decreases the levels of proinflammatory transcription factors and increases the levels of anti-inflammatory ones.

To examine the potential indirect effect of tuftsin on T cells, we designed culture conditions to mimic the effects of *in vivo* tuftsin infusion in the EAE model. As it has been previously described that early excitotoxic injury plays a crucial role in EAE [40], [41], we simulated this *in culture* by treating primary neurons overnight with 100 µM glutamate to generate neuronal-conditioned medium (NCM). Primary microglia were then incubated with the NCM and tuftsin for 10 hours to generate the NCM-treated microglial-conditioned medium, which was collected and added to primed T cells. 3 days later, supernatants from the T cell cultures were collected and analyzed as above. Microglial-conditioned medium prepared from microglia stimulated only with NCM (NCM curve, 0 µg/ml concentration point) had no effect on cytokine secretion by the T cells (Fig. 1B,C) in comparison to medium prepared from non-stimulated microglia (tuftsin curve, 0 µg/ml concentration point). Microglial-conditioned medium prepared from microglia stimulated only with tuftsin also resulted in unchanged cytokine secretion by the T cells. However, since tuftsin had a direct effect on T cells to stimulate TNFαlpha production (Fig. 1A), it would appear that tuftsin-stimulated microglia do secrete factors that counter the pro-inflammatory T cell response to tuftsin. More dramatically, the combination of NCM and tuftsin stimulation of microglia yielded a microglial-conditioned medium that had a profound effect on the TNFα and IL10 levels recorded. TNFα release decreased 4-fold in a tuftsin dose-dependent manner, whereas IL10 release increased nearly 10-fold, resulting in a >35-fold shift towards immunoprotection. Taken together, these *in culture* experiments suggested that modulating microglial activity with tuftsin under glutamate-stimulatory conditions affects the T cell phenotype by down-regulating inflammatory Th1 responses while up-regulating cytokines known to have immunosupressive effects consistent with a Th2 phenotype.

### Tuftsin-activated microglia exposed to excitotoxic media polarize to an anti-inflammatory, M2 phenotype

As microglial involvement is necessary for mediating tuftsin's beneficial effects *in culture*, we next analyzed the changes that occur in the treated microglia. Similar to T cells, microglia have pro- and anti-inflammatory subsets known as M1 and M2. M1 microglia, which are neurodegenerative in a model of spinal cord injury, produce TNFα and nitric oxide, while neuroprotective M2 microglia release IL10 and TGFβ [42]–[44]. Additionally, administration of M2 monocytes in EAE suppressed ongoing severe symptoms [45]. We proposed that the combination of tuftsin and NCM would cause microglia to shift to an anti-inflammatory, M2 phenotype, which could explain the prevalence of Th2 cytokines in the previous figure.

**Figure 2:**
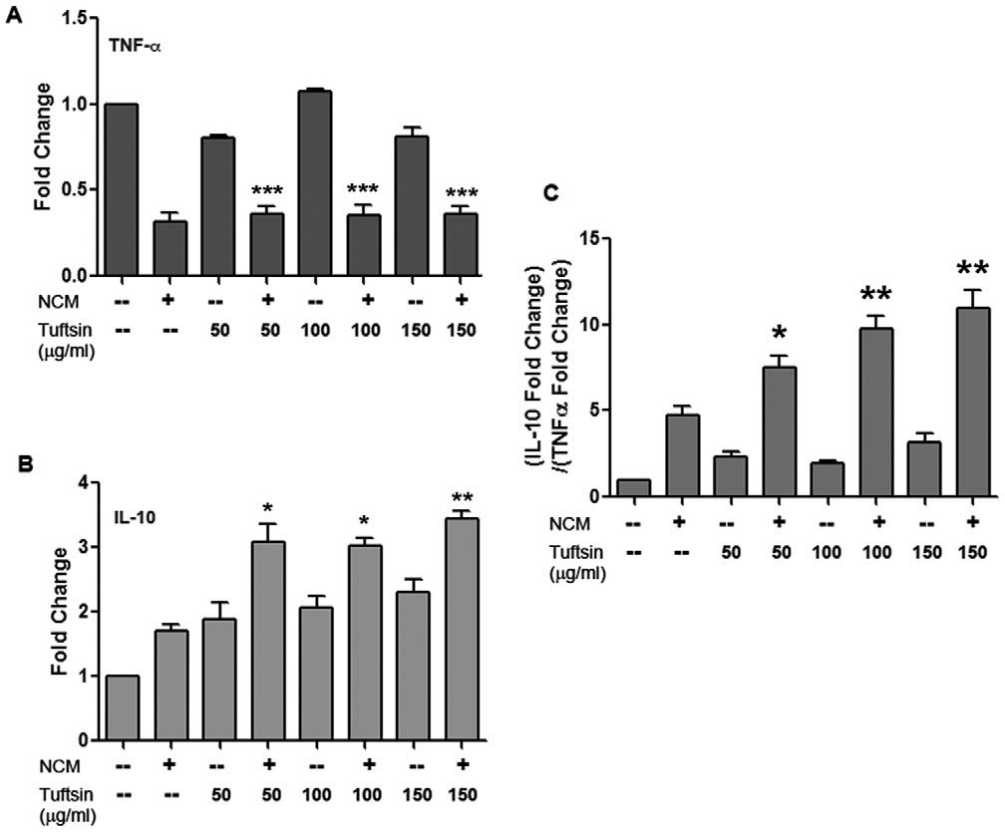
Treatment of microglia with tuftsin and excitotoxic media causes an M2 shift. Quantitative RT-PCR was performed to analyze changes in gene expression of the M1 marker TNFα (**A**) and the M2 marker IL10 (**B**) in response to tuftsin and NCM treatment for 10 hours. Primary microglia were treated with increasing concentrations of tuftsin or tuftsin and NCM prepared from primary cortical neurons exposed to 100 µM glutamate overnight. (**C**) The ratio of the fold change of IL10 (M2) to the fold change of TNFα (M1). n = 3, *, p<0.5; **, p<0.01; ***, p<0.001. In (**A**) and (**B**) the comparisons are between tuftsin and tuftsin/NCM at each concentration. In (**C**) the comparisons are between NCM and tuftsin/NCM.

To observe the effect of tuftsin and NCM on the microglial subtype, we treated primary microglia for 10 hours with NCM and increasing concentrations of tuftsin as above. From these cells we harvested RNA and performed quantitative real-time PCR to determine microglial phenotype based on TNFα levels for M1, and IL10 levels for M2. The combination of NCM and tuftsin at all concentrations significantly affected M1 and M2 specific genes in the microglia, with a 3-fold increase in IL10 and a 3-fold decrease in TNFα (Fig. 2A,B). More dramatically, when observing the ratio of the change in M2 to M1 genes, there is greater than a 10-fold shift towards neuroprotection (Fig. 2C). Surprisingly, NCM alone shows a slight shift towards neuroprotection, with a decrease in TNFα and an increase in IL10 gene expression. However, the combination of tuftsin and NCM shows a significantly higher upregulation of IL10 than NCM alone, and the degree of neuroprotection represented by the M2/M1 ratio for NCM alone is significantly less than the combination of NCM and tuftsin. Additionally, it has been shown that moderate neuronal cell death can cause microglia to assume a more protective phenotype, which could explain this phenomenon [46]. Thus, these experiments indicate that the combination of NCM and tuftsin promotes an anti-inflammatory, M2 phenotype in microglia, which is the likely cause for the polarization of T cells towards a Th2 phenotype as shown above.

#### Microglia shed factors in response to tuftsin treatment

To determine what components present in the microglial conditioned medium could act on T cells, we repeated the experiment described in Fig. 1 and 2, collected and concentrated media from treated microglia. The control microglial conditioned media, which was derived from microglia exposed to NCM alone (MgCM), and samples obtained from microglia treated with NCM and tuftsin (MgCM+tuftsin) were concentrated and digested with trypsin and subjected to shotgun proteomic analysis. Several proteins were identified whose expression was upregulated with tuftsin treatment, a short list of which is shown (Table 1). The presence of some of these factors (e.g., PLD3, Nrp1) were confirmed by western blot analysis (Figure S1). Most interestingly, Nrp1, tuftsin's receptor [47], was upregulated in the MgCM+Tuf treatment. The presence of this factor in the analyzed conditioned media, despite its role as a cell surface receptor, is likely due to its shedding from the cell surface by the protease ADAM10 [48], which was also present in the analyzed conditioned media.

**Table 1:** Treatment of microglia with NCM and tuftsin induced the release of immunomodulatory factors.

Description	Levels in MgCM	Levels in MgCM+tuf	MgCM+tuf/MgCM
Uncharacterized protein Igf1	1	8	8
Uncharacterized protein TnfaIp8	1	8	8
Phospholipase D3	2	10	5
Glia maturation factor Gmfb	2	9	4.5
Mannosidase alpha 1C	1	4	4
Procathepsin H	6	22	3.7
Monocyte differentiation antigen CD14	8	26	3.3
Macrophage scavenger receptor	1	3	3
Neuropilin2	1	3	3
Neuropilin1	10	13	1.3
Disintegrin and metalloproteinase domain-containing protein 10, Adam10	0	4	>4

To characterize the factors released from microglia under EAE-like conditions, microglia were treated for 10 hours with NCM isolated from neurons undergoing excitotoxic injury in the presence or absence of tuftsin. Concentrated conditioned media were analyzed by multidimensional chromatography and tandem mass spectrometry. Partial list of the secreted proteins is shown. Spectral counts (MS/MS) of proteins identified from the media of microglia treated with NCM alone (MgCM) or a combination of NCM and 100 µg/ml of tuftsin (MgCM+Tuf) for 10 hours are listed in the respected columns. Ratios derived from spectral counts were used to predict the trend of differential expression.

### The T cell shift towards immunosupression after exposure to excitotoxic- and tuftsin-activated microglial conditioned medium takes place on a transcriptional level

We extended these observations by performing RT-PCR on mRNA from T cells cultured in differing types of conditioned medium, using an array of primers to analyze a panel of cytokines and their receptors. Reinforcing the findings shown in Fig. 1B,C, the mRNA expression level of TNFα was decreased 9-fold, and that of IL10 increased 23-fold, when T cells were cultured in conditioned medium prepared from microglia stimulated by both NCM and tuftsin (Tables 2 and 3). This result links the changes in cytokine secretion to the altered levels of inflammation- and immunosuppression-promoting transcription factors that we had reported previously [21].

**Table 2:** PCR array results indicating changes in mRNA levels of T cells after tuftsin treatment.

	Fold Change in mRNA levels of anti-inflammatory cytokines and receptors (compared to control group)		
Gene Symbol	Group 1 (NCM)	Group 2 (Tuftsin)	Group 3 (Tuftsin+NCM)
Tumor necrosis factor (TNF)α	1.38	1.0	0.12
TNF receptor 1a (TNFRSF1a)	0.77	0.75	0.12
TNFRSF1b	1.27	0.96	0.09
Bcl6	0.89	0.86	0.20

T cells were treated with MgCM that was isolated from microglia treated with NCM alone, 100 µg/ml of tuftsin alone, or a combination of NCM+100 µg/ml tuftsin for 10 hours. After 3 days, mRNA was isolated from T cells and was reverse-transcribed and used to probe a PCR array kit of inflammatory cytokines and their receptors to determine the subtype of T cells present as a result of treatment, as described in Methods. n = 2.

**Table 3:** PCR array results indicating changes in mRNA levels of T cells after tuftsin treatment.

	Fold Change in mRNA levels of pro-inflammatory cytokines and receptors (compared to control group)		
Gene Symbol	Group 1 (NCM)	Group 2 (Tuftsin)	Group 3 (Tuftsin+NCM)
Interleukin 10 (IL10)	2.54	1.03	23.42
IL10 receptor (IL10Ra)	0.84	0.70	19.42
IL10Rb	1.09	1.19	6.59
IL13	1.06	0.77	12.73
IL4	1.67	0.79	15.12
Transforming growth factor (TGF) beta 1	1.30	1.24	27.61
Chemokine receptor (CCR)3	0.97	0.84	36.83
CCR5	0.93	31.09	86.96
Interferon (IFN) gamma	1.19	0.84	7.61

T cells were treated with MgCM that was isolated from microglia treated with NCM alone, 100 µg/ml of tuftsin alone, or a combination of NCM+100 µg/ml tuftsin for 10 hours. After 3 days, mRNA was isolated from T cells and was reverse-transcribed and used to probe a PCR array kit of inflammatory cytokines and their receptors to determine the subtype of T cells present as a result of treatment, as described in Methods. n = 2.

Expanding the response pattern, the mRNA levels of TNFalpha receptors 1a and 1b also decreased after treatment with NCM plus tuftsin-conditioned microglial medium, as was the case for Bcl6, which has been described to suppress Th2 responses [49]. Conversely, the levels of expression of the αlpha and beta receptors for IL10 increased, as did those for several other anti-inflammatory cytokines such as Interleukin 13 (IL13), Interleukin 4 (IL4), and Transforming Growth Factor β1 (TGFβ1), which stimulates the development of immunosuppressive regulatory T cells [50]. Similarly, CCR3, a chemokine receptor that mediates recruitment of Th2 cells [51], was upregulated.

CCR5, a chemokine receptor expressed primarily by Th1 cells [52], was the only gene that responded significantly to medium conditioned only with tuftsin, suggesting that it potentially ensues from direct T cell or microglial stimulation by tuftsin rather than by factors produced by the microglia in the context of excitotoxicity. Finally, modest upregulation was also observed for interferon γ (IFNγ), which has been reported to function in both pro- and anti-inflammatory contexts in EAE, as IFN-γ knockout mice exhibit increased susceptibility to EAE [53]. Taken together, the PCR array results revealed that modulating microglia activity with NCM and tuftsin efficiently down-regulates Th1-type responses, upregulates Th2-type responses, and releases factors that promote immunosuppressive regulatory T cell formation.

### The transcriptional changes observed in T cells exposed to excitotoxic- and tuftsin-activated microglial conditioned medium are mediated by suppression of STAT1-signaling and stabilization of the FoxP3 transcription factor

**Figure 3:**
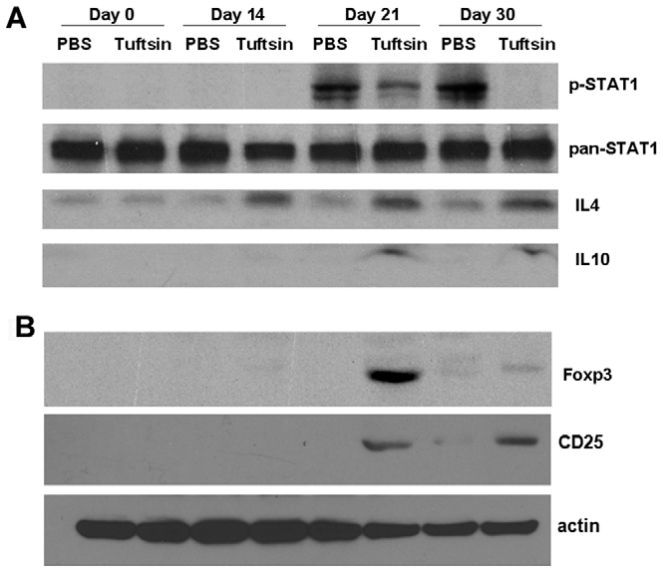
Tuftsin infusion induces the expression of Th2 and Treg cell markers. Western blot analyses were performed on mouse spinal cord extracts at different timepoints following EAE induction to examine protein expression in the presence or absence of 500 µM tuftsin infusion. (**A**) Phosphorylation of STAT1 and the levels of the anti-inflammatory cytokines IL10 and IL4 were assessed in extracts from tuftsin-infused wt mice subjected to EAE. Equal loading of protein was confirmed by pan-STAT1 blotting. (**B**) Levels of the Treg transcription factor FoxP3 and the Treg cell surface marker CD25. Equal loading of protein was confirmed by actin blotting. (Western blots were repeated three times on mouse spinal cord samples from three separate EAE experiments, n = 3).

We next assessed regulatory pathways potentially involved in the transcriptional changes seen in Tables 2 and 3. STAT1 is a member of the Signal Transducers and Activators of Transcription family of transcription factors that upregulates pro-inflammatory cytokines (TNFα and IL12) while repressing production of anti-inflammatory cytokines (IL10). STAT1 phosphorylation (activation) has been shown to be increased in EAE lesions [54]. In contrast, FoxP3, a regulatory T cell transcription factor, facilitates the commitment towards regulatory T cell lineage by amplifying and stabilizing its own expression and repressing alternative cell fates in response to the immunosuppressive cytokine TGFβ, which suppresses STAT1 signaling [50] and upregulates the transmembrane surface protein CD25. We evaluated the levels of STAT1 phosphorylation, FoxP3, CD25, and immunosuppressive cytokine expression in spinal cord homogenates prepared from mice subjected to MOG-induced EAE with and without administration of tuftsin. As shown in Fig. 3, phospho-STAT1 expression increased on days 21 and 30 in MOG-immunized PBS-infused control mice, and levels of the immunosuppressive cytokines IL4 and IL10, FoxP3, and the Treg marker CD25 remained low or undetectable. However, administration of tuftsin strongly blunted the increase in phospho-STAT1 while increasing synthesis of IL10, IL4, FoxP3, and CD25. Thus, tuftsin administration shifts the balance of the immune response from pro-inflammation (phospho-STAT1) to anti-inflammation (IL4 and IL10) and immunosuppression (FoxP3, CD25). Taken together, these results suggest that *in vivo*, tuftsin infusion promotes downregulation of Th1 signaling, increased activity of Th2 cells, and expansion of Treg cells.

### T cells stimulated by excitotoxic- and tuftsin-activated microglial conditioned medium reverse clinical symptoms following adoptive transfer into mice with established EAE

**Figure 4:**
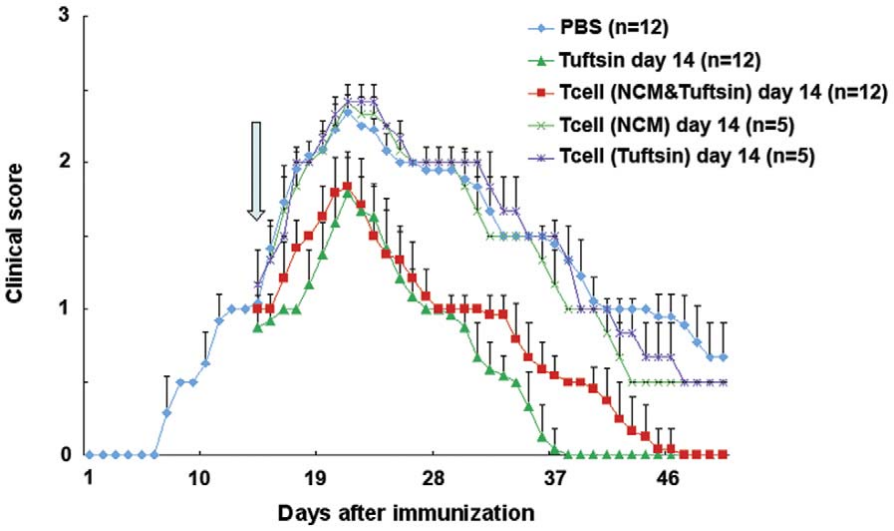
Adoptive transfer of tuftsin and NCM modulated T cells on day 14 ameliorates established EAE. Wild-type (WT) mice were injected with MOG_35–55_ peptide in CFA and pertussis toxin to induce EAE. Osmotic pumps filled with PBS or 500 µM tuftsin in PBS were implanted in the back of the mice on day 1 after MOG immunization. 5×10^6^ T cells, modulated as follows: tuftsin/NCM-treated T cells; tuftsin-only treated T cells; NCM-only treated T cells, were injected intravenously into recipient C57BL/6 mice immunized with MOG 14 days after the onset of disease (arrow indicates timepoint when T cell transfer was performed) and the disease severity scored as described in Methods. (The experiment was repeated twice. Cumulative data are provided; total number of animals tested, n = 12/PBS-infused mice; n = 12/tuftsin-infused mice; n = 12/mice adoptively transferred with tuftsin plus NCM-modulated T cells; n = 5/mice adoptively transferred with tuftsin-only treated T cells; n = 5/mice adoptively transferred with NCM-only treated T cells). Statistical analysis was performed, as described in the Methods. All time points between tuftsin/NCM-treated T cells and PBS control were found to be significant (p<0.001) except for days 14–15, 34 and 41–44). All the timepoints between tuftsin alone and PBS were significant (p<0.001). No difference was evident between tuftsin and tuftsin/NCM-treated T cells.

To assess whether the protective effect of tuftsin in EAE is mediated by indirect modulation of the balance and activity of T cell subsets, we transferred T cells cultured in excitotoxic- and tuftsin-activated microglial conditioned medium into mice that had been subjected to MOG immunization to generate EAE. 5×10^6^ T cells cultured in excitotoxic- and tuftsin-activated microglial conditioned medium as described above were injected intravenously 14 days after the onset of disease into recipient C57BL/6 mice immunized with MOG. The treatment was initiated on day 14 to assess whether existing EAE could be modified. T cells treated with NCM/tuftsin-modulated microglial medium decreased the clinical signs of EAE, and the disease course paralleled that of tuftsin-infused animals (Fig. 4). In contrast, T cells treated with NCM-only or tuftsin-only stimulated microglial conditioned medium were unable to alter the clinical course of EAE in comparison to that of PBS-infused control mice. The results suggest that the shift towards immunosuppression observed for T cells after culture in excitotoxic- and tuftsin-activated microglial conditioned medium is functional in vivo and just as potent as directly administering tuftsin to the EAE-afflicted mice. Taken together, this suggests that the mechanism of tuftsin's suppression of EAE *in vivo* is via promotion of a shift in the T cell phenotype consistent with suppression of the immune response.

### EAE mice adoptively transferred with T cells stimulated by excitotoxic- and tuftsin-activated microglial conditioned medium have attenuated demyelination

**Figure 5:**
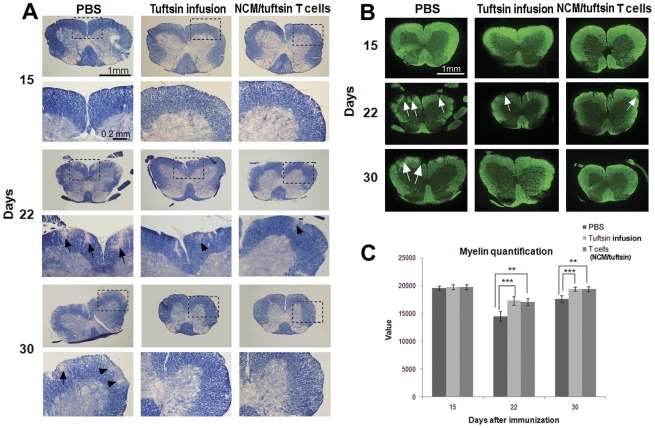
Attenuated demyelination in EAE mice adoptively transferred with tuftsin/NCM-modulated T cells. (**A**) Frozen cross-sections of spinal cords were isolated at different time points during the EAE from PBS-infused wild-type mice, Tuftsin-infused wild-type mice, and wild-type mice adoptively transferred with Tuftsin/NCM-modulated T cells. Spinal cord sections were stained with Eriochrome cyanine (EC), which visualizes myelin (blue). Sites of demyelination are indicated by arrows. (**B**) Use of FluoroMyelin to fluorescently and **C**. quantitatively image myelin. Quantifications were performed on five sections per mouse for three different mice per timepoint in two separate EAE experiments; results are presented as an average with error bars indicating the standard error of the mean. A two-tailed t test was performed to analyze the significance of the difference of FluoroMyelin staining intensity across groups at each timepoint (*** p<0.001; **p<0.01).

The histological hallmark of EAE consists of demyelination, which can be assessed using Eriochrome Cyanine (EC) staining. The timing of spinal cord demyelination is well established to correlate closely with clinical progression in this disease model. MOG-induced EAE was established in three groups of mice, which were then individually infused with PBS, infused with tuftsin, or adoptively transferred with T cells stimulated by excitotoxic- and tuftsin-activated microglial conditioned medium as above. None of the groups exhibited significant demyelination on day 15 (Fig. 5A). By the peak of disease (day 22), however, the PBS-infused EAE mice were remarkable for they showed severe demyelination in the spinal cord white matter, as characterized by large areas devoid of EC staining (arrows). In contrast, EAE mice infused with tuftsin exhibited much less demyelination, and mice adoptively transferred with the stimulated T cells were similarly protected. At day 30, there were still apparent demyelinated areas visible in the PBS-infused mice (arrows), but the two treated groups had undergone almost complete remyelination.

Demyelination can also be evaluated by FluoroMyelin, a fluorescent myelin stain, with which similar results were obtained (Fig. 5B, arrows indicate sites of demyelination). Quantification of the fluorescence intensities (Fig. 5C) revealed that the extent of demyelination in the group of mice that received the adoptively transferred T cells was comparable to that observed in the tuftsin-infused mice group, and both showed significantly increased myelination than the group of mice infused with PBS.

### Adoptive transfer of stimulated T cells decreases microglia/macrophage infiltration and activation in mice subjected to EAE

**Figure 6:**
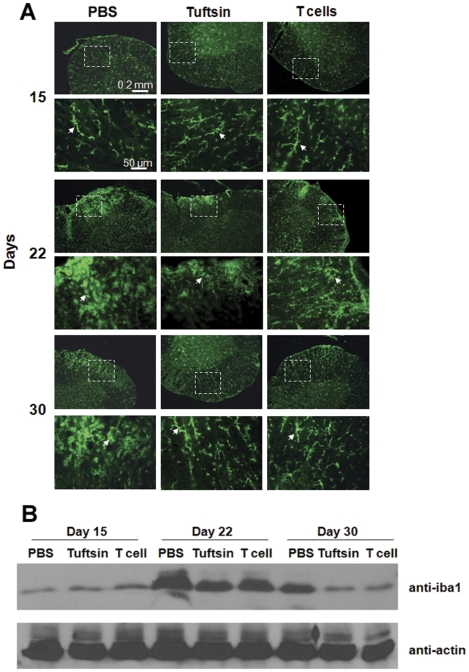
Reduced microglia/macrophage infiltration and activation in mice with adoptive transfer. (**A**) Frozen cross-sections of spinal cords from PBS, tuftsin-infused and T-cell adoptively transferred mice at different time points in EAE were stained with anti-Iba1 to detect microglia/macrophages. Iba1 is expressed on both resting and activated microglia/macrophages, which can be distinguished by their morphological appearance. Arrows indicate resting microglia, characterized by long, thin cell bodies. Arrowheads indicate activated microglia/macrophages that are characterized by thicker, rounded cell bodies and multi-branched cellular protrusions (The experiment was repeated three times. In total, five mice per genotype were assessed for immunostaining at each timepoint; representative data are provided.) (**B**) Mouse spinal cord extracts were collected at different timepoints after induction of EAE and analyzed using western blotting to quantify the levels of Iba1. Equal loading of protein was confirmed by anti-actin blotting (the western blot was repeated three times on mouse spinal cord samples from two separate EAE experiments).

During EAE, activated microglia signal to attract infiltrating T cells which attack the endogenous myelin. Myelin degradation products then recruit additional activated microglia to the demyelinated areas to remove myelin and cellular debris using their phagocytotic capacity [55], [56]. We evaluated the levels of microglia/macrophage activation and recruitment at different time-points (Day 15, 22, 30) during EAE, using an antibody against Iba1, which is expressed at low levels by resting microglia/macrophages and becomes upregulated during their activation. Iba1 staining on day 15 revealed resting microglia/macrophages in all groups, as defined by the cells' ramified morphology with long thin cell bodies (arrows, Fig. 6A). On day 22, PBS-infused mice exhibited large numbers of microglia/macrophages that had undergone activation in the white matter of the spinal cord, as indicated by their ameboid shape and extensive branches, and the activated microglia/macrophages continued to be evident through day 30. In contrast, in tuftsin-infused mice and in mice with adoptively transferred T cells, there were also activated microglia by day 22, but they were much less frequent than in the PBS-infused mice. By day 30, the number of Iba1-positive cells had decreased in the treated animals, with most of the microglia and macrophages re-assuming a resting morphology.

Spinal cord extracts were assayed in parallel using western blotting to quantitate Iba1 expression, as a semi-quantitative measure of the extent of microglia/macrophage recruitment and activation (Fig. 6B). All three groups expressed Iba1 at basal levels on day 15, and the level of Iba1 increased as disease progressed. PBS-infused animals showed a dramatic upregulation of Iba1 levels on day 22, and then decreased while still remaining elevated by day 30. In contrast, tuftsin-infused mice and mice that received NCM/tuftsin treated T cells exhibited blunted Iba1 upregulation on day 22, and had returned to basal levels of expression of by day 30. These results correlate with the extent of microglia/macrophage activation described in panel A and with the clinical symptoms described in Fig. 4 and histological features shown in Fig. 5.

## Discussion

Tuftsin's broad activities on phagocytotic cells, especially microglia and macrophages, make the peptide a potential candidate for immunotherapy. As evidence, tuftsin and its analogs have been chemically synthesized and applied in a variety of clinical studies [57]. As the normal tuftsin serum level is 250–500 ng/ml and the LD_50_ dose of tuftsin is 2.4 mg/ml, a serum level of 60 µg/ml is a suitable physiological concentration for applying tuftsin in therapeutic studies. Here, we delivered tuftsin through mini-osmotic pumps at a concentration of 500 µM, with an infusion rate of 0.25 µl/hour. In theory, an infusion of tuftsin at 0.06 µg per hour will diffuse along the length of the spinal cord and surrounding tissues, bringing it to a physiological concentration for the therapeutic effects without other untoward effects.

In our previous work, we reported that early activation of microglia/macrophages by tuftsin abrogated clinical symptoms in EAE mice, and this correlated with a switch towards increased expression of a Th2 transcription factor. These findings led us to explore the concept of protective autoimmunity. In MS/EAE, the balance between the pro-inflammatory Th1 response and the anti-inflammatory Th2 response, together with the beneficial effect of the regulatory T cells, is thought to determine the outcome of MS/EAE. Here, we have found that after tuftsin treatment, markers of Th2 and Treg cells are upregulated, suggesting that there is activation of Th2 cells and expansion of Tregs. Our current studies confirmed the idea that early activation of microglia by tuftsin results in a coordination of the immune response that favors protective autoimmunity as opposed to autoimmune disease.

However, it is not only T cells that function as the mediators of tuftsin's effects, as microglia are equally responsible for promoting disease progression or resolution. Microglia have been shown to play an important role in the demyelination process, both through the induction of the inflammatory cascade by releasing pro-inflammatory cytokines [44], and through interactions with MBP. In the CNS, tissue plasminogen activator recruits and activates microglia, which then promote demyelination through contact with MBP peptides in the myelin sheath [58], [59]. On the other hand, microglia have been shown to play important roles in the remyelination process, both through phagocytosis of debris and support of myelinating oligodendrocytes [60], as well as through promotion of axonal regeneration [61]. From the data presented here, it is likely that the dual roles of microglia in the myelination process can also be explained by the presence of M1 and M2 microglial subsets; as we show tuftsin is capable of promoting the M2 anti-inflammatory phenotype in EAE.

Many treatments for MS aim at repression of the immune response. Some reagents work by inhibiting activation of microglia and T cells and cause a downregulation of the production of proinflammatory cytokines [62]. These treatments attenuate MS symptoms through their “anti-inflammatory effects.” Tuftsin, however, appears to affect MS/EAE symptoms in a different manner, by shifting the immune response towards an anti-inflammatory phenotype. Tuftsin appears to modulate T cell behaviors by enhancing Th2 cytokines and expanding Tregs, not by just inhibiting the Th1 response. Thus tuftsin exerts its effect through a more broad-based “modulation of the immune system.”

As we have shown above, microglial conditioned media treated with NCM alone or tuftsin alone on T cells was not capable of promoting the shift towards anti-inflammation as the combination treatment does. These cells appear to need a “two-hit” treatment to successfully convert to a protective phenotype. This finding may explain why NCM alone can promote a slight anti-inflammatory shift in microglial populations that is not recapitulated downstream in T cells, for there could be some other factors in play that NCM alone is incapable of upregulating. However, the nature of this second signal induced by tuftsin treatment is currently unknown. Our proteomic screen identified several factors shed in the media in the presence of tuftsin. Some of these factors were common for the activated microglial secretome [63], while others were unique to the tuftsin treatment.

Despite its 30-year history, the mechanism of action of tuftsin remain unknown. However, based on the broad range of action of tuftsin in both the immune and nervous systems, it is expected that the tuftsin receptor(s) should be common in both systems. Cultured human aortic and umbilical vein endothelial cells were shown to express tuftsin receptors and that the identity of the receptor is neuropilin-1 [47]. Because neuropilin-1 plays critical roles in the immune, vascular, and nervous systems and interacts with a number of different ligands, cell surface receptors and adhesion proteins [64]–[66], it is possible that some of the previously reported effects of tuftsin are mediated through neuropilin-1, which could function as the second signal of the “two-hit” treatment that is necessary to promote a full shift towards neuroprotection.

## Supporting Information

Figure S1 **Microglial shed factors in conditioned media.** To confirm the results of the proteomic screen aiming to determine what factors are released by stimulated microglia, conditioned media from primary microglia treated with NCM alone or a combination of NCM and 100 µg/ml tuftsin for 10 hours were concentrated and analyzed by western blot for the abundance of PLD3 (A) or Nrp1 (B). This western blot experiment was repeated three times on media isolated from three separate *in vitro* experiments. PLD3 fold change: 5.37 (5-fold change documented from the proteomic data), Nrp-1 fold change: 1.23 (1.3 in the proteomic data).
(TIF)
