# Supplementary figures and images for: Tuftsin Promotes an Anti-Inflammatory Switch and Attenuates Symptoms in Experimental Autoimmune Encephalomyelitis
Source: PLoS One. 2012 Apr 17;7(4):e34933. doi: 10.1371/journal.pone.0034933 (PMC3328491; doi:10.1371/journal.pone.0034933)

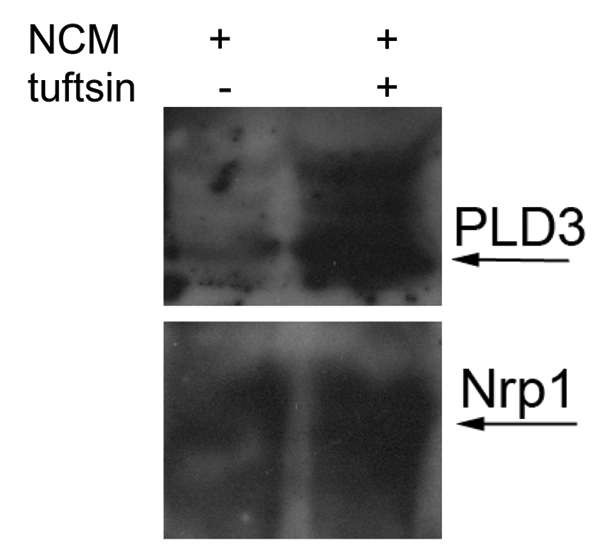

Supplement: Figure S1 — Microglial shed factors in conditioned media. To confirm the results of the proteomic screen aiming to determine what factors are released by stimulated microglia, conditioned media from primary microglia treated with NCM alone or a combination of NCM and 100 µg/ml tuftsin for 10 hours were concentrated and analyzed by western blot for the abundance of PLD3 (A) or Nrp1 (B). This western blot experiment was repeated three times on media isolated from three separate in vitro experiments. PLD3 fold change: 5.37 (5-fold change documented from the proteomic data), Nrp-1 fold change: 1.23 (1.3 in the proteomic data). (TIF) [file pone.0034933.s001.tif]
